# Dying in the Intensive Care Unit (ICU): A Retrospective Descriptive Analysis of Deaths in the ICU in a Communal Tertiary Hospital in Germany

**DOI:** 10.1155/2020/2356019

**Published:** 2020-03-01

**Authors:** Esma Ay, Markus. A. Weigand, Rainer Röhrig, Marco Gruss

**Affiliations:** ^1^Department of Anaesthesiology, Intensive Care Medicine and Pain Therapy, Klinikum Hanau GmbH, Leimenstrasse 20, Hanau D-63450, Germany; ^2^Department of Anaesthesiology, Heidelberg University Hospital, Im Neuenheimer Feld 110, Heidelberg D-69120, Germany; ^3^Department of Medical Informatics, University Hospital RWTH Aachen, Aachen, Germany

## Abstract

**Background:**

Modern intensive care methods led to an increased survival of critically ill patients over the last decades. But an unreflected application of modern intensive care measures might lead to prolonged treatment for incurable diseases, and an inadaequate or too aggressive therapy can prolong the dying process of patients. In this study, we analysed end-of-life decisions regarding withholding and withdrawal of intensive care measures in a German intensive care unit (ICU) of a communal tertiary hospital.

**Methods:**

Patient datasets of all adult patients dying in an ICU or an intermediate care unit (IMC) in a tertiary communal hospital (Klinikum Hanau, Germany) between 01.01.2011 and 31.12.2012 were analysed for withholding and withdrawal of intensive care measures.

**Results:**

During the two-year period, 1317 adult patients died in Klinikum Hanau. Of these, 489 (37%) died either in an ICU/IMC unit. The majority of those deceased patients (*n* = 427, 87%) was 60 years or older. In 306 (62%) of 489 patients, at least one life-sustaining measure was withheld or withdrawn. In 297 (61%) of 489 patients dying in ICU/IMC, any type of therapy was withheld, and in 139 patients (28%), any type of therapy was withdrawn. Mostly, cardiopulmonary resuscitation (*n* = 427, 87%) was 60 years or older. In 306 (62%) of 489 patients, at least one life-sustaining measure was withheld or withdrawn. In 297 (61%) of 489 patients dying in ICU/IMC, any type of therapy was withheld, and in 139 patients (28%), any type of therapy was withdrawn. Mostly, cardiopulmonary resuscitation (*n* = 427, 87%) was 60 years or older. In 306 (62%) of 489 patients, at least one life-sustaining measure was withheld or withdrawn. In 297 (61%) of 489 patients dying in ICU/IMC, any type of therapy was withheld, and in 139 patients (28%), any type of therapy was withdrawn. Mostly, cardiopulmonary resuscitation (*n* = 427, 87%) was 60 years or older. In 306 (62%) of 489 patients, at least one life-sustaining measure was withheld or withdrawn. In 297 (61%) of 489 patients dying in ICU/IMC, any type of therapy was withheld, and in 139 patients (28%), any type of therapy was withdrawn. Mostly, cardiopulmonary resuscitation (*n* = 427, 87%) was 60 years or older. In 306 (62%) of 489 patients, at least one life-sustaining measure was withheld or withdrawn. In 297 (61%) of 489 patients dying in ICU/IMC, any type of therapy was withheld, and in 139 patients (28%), any type of therapy was withdrawn. Mostly, cardiopulmonary resuscitation (*n* = 427, 87%) was 60 years or older. In 306 (62%) of 489 patients, at least one life-sustaining measure was withheld or withdrawn. In 297 (61%) of 489 patients dying in ICU/IMC, any type of therapy was withheld, and in 139 patients (28%), any type of therapy was withdrawn. Mostly, cardiopulmonary resuscitation (

**Conclusions:**

About one-third of patients dying in the hospital died in ICU/IMC. At least one life-sustaining therapy was limited/withdrawn in more than 60% of those patients. Withholding of a therapy was more common than active therapy withdrawal. Ventilation and renal replacement therapy were withdrawn in less than 5% of patients, respectively.

## 1. Introduction

The developments in modern medicine and especially in intensive care medicine after the Second World War led to an increased survival of critically ill patients. Over the last decades, there was an increased demand for intensive care unit (ICU) beds and an increasing number of hospitalised patients admitted to an intensive care unit during their hospital stay [1]. While more and more people survive acute critical situations, the developments in the organ support can lead to a state called “chronic critical illness” [2]. Because people are getting older and older, it can be expected that much more patients will need intensive care support during their hospital stay. In Germany, with an overall population of about 80 million people in 2014, 27%/6% of people were 65/80 years or older, and it is expected that these fractions will increase to 38% and 13% in 2050, respectively [3]. The numbers for the European Union are very similar [3]. An unreflected application of intensive care measures might lead to prolonged treatment for incurable diseases, and an inadequate or too aggressive therapy can prolong the dying process [4, 5]. Due to the increasing number of patients dying in ICUs [1], there has been a lot of discussion about end-of-life decisions in intensive care units and how much and what intensity of care is in patient's best interest [4, 6–13]. Nowadays, withholding or even withdrawal of intensive care measures is a common practice in many ICUs, and several intensive care medicine societies have pointed out recommendations regarding end-of-life decisions [14–17].

Several studies investigated end-of-life decisions in different countries and different regions of the world. Similarities but also great variabilities in withholding and withdrawal practices have been described depending on socioeconomic, cultural, and religious background [1, 18–21].

Whereas most studies investigating end-of-life decisions focused on university hospitals [22, 23], there are less data about therapy withholding or withdrawal in community or teaching hospitals [24]. In Germany, there is only limited knowledge about the place of death and almost no data about where and especially under what circumstances patients are dying in hospitals [25].

In this study, we explored withholding and withdrawal of life-sustaining therapy in an 750-bed tertiary community and teaching hospital in detail by retrospective analysis of all patients dying in an ICU or intermediate care unit (IMC) in 2011 or 2012.

## 2. Materials and Methods

In this retrospective explorative study, we looked at end-of-life practices in adult patients who died in *Klinikum Hanau*, a communal German tertiary hospital with ∼750 beds including 20 ICU and 16 IMC beds. Ethical approval was given before analysing data by the Landesärztekammer Hessen (FF 131/2013, 23.01.2014). We analysed charts of adult patients who died in an ICU or an IMC between 01.01.2011 and 31.12.2012 regarding any withholding or withdrawal of life-sustaining therapies. Originally, patients who died in 2010 were planned to be analysed but had to be excluded because patients' charts were archived in hardcopy form at an external location and therefore could not be looked at in an acceptable time course.

Patient charts of patients who died in 2011 or 2012 were archived in an electronic form. Any chart of these patients was looked at and analysed for patients' master data, date and time of admission to hospital and last admission to ICU, date and time of death, existence of an advance directive, life-sustaining measures during time of death, and if and which measures of life-sustaining therapy were withheld or withdrawn. Therapy was defined as withheldif decision was made not to start or to increase at least one life-sustaining intervention [19] and defined as withdrawn if decision was made to actively stop a life-sustaining intervention presently being given [19]. All 2011/2012 patients' charts were analysed by one investigator (E. A.), double-checked by the second investigator (M. G.), and discussed until an agreement was achieved. Only cases with clear documentation or unambiguous hints for withholding or withdrawal, e.g., stop of catecholamine infusion prior to death, were classified as withheld or withdrawn. All other cases were classified as negative. If there was no clear documentation regarding withholding of therapy but this could be reasoned by indirect hints in the patient's chart, it was classified as “not explicitly mentioned.”

As far as possible, data were extracted from the hospital information system (SAP®, SAP Deutschland SE and Co. KG, Germany). The time interval from last admission on IMC/ICU until death was calculated, and the time interval from either withholding or withdrawal of therapy until death was estimated as exact as possible and classified in daily intervals. A more exact definition of the withholding/withdrawal “time point” was not possible mainly due to paper-based documentation. The patient's main medical problem was classified according to the main classification in the German diagnosis-related group (DRG) system. All drugs not belonging to catecholamines, analgesics/sedatives, antibiotics, or feeding/nutrition were classified as “other drugs.”

Graded or nominal classified variables are shown in absolute and relative frequency. Correlations data were analysed in cross tables by Chi-square and exact Fisher's test. Most variables were analysed descriptively by mean, median, and standard deviation. Differences between two groups (e.g., two groups of different ages) were tested by *t*-test or variance analysis, if more than two groups were compared. SPSS®, Origin®, and R® were used for data analysis.

## 3. Results

**Figure 1:**
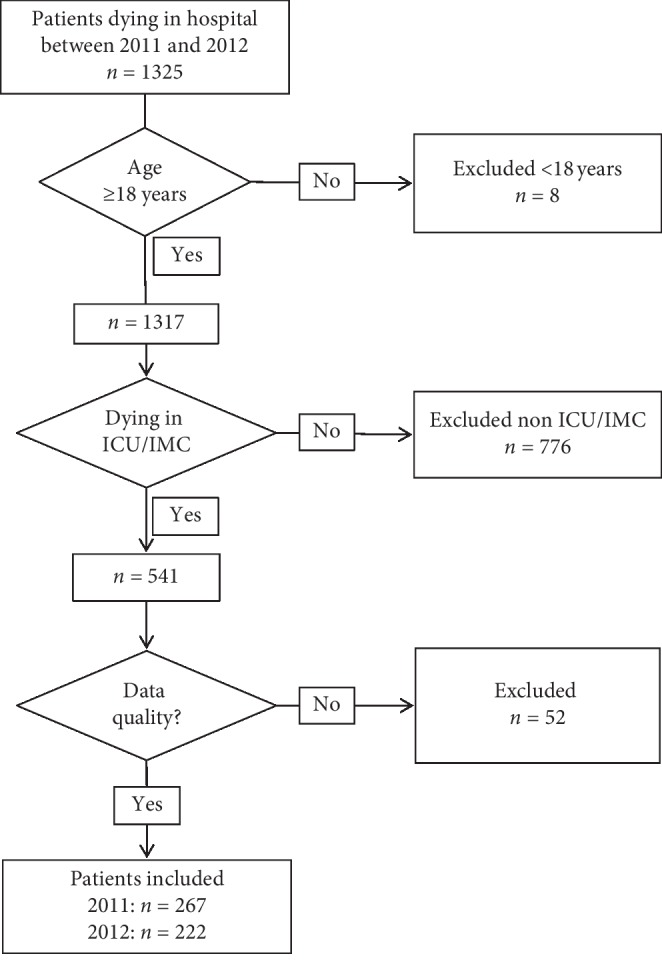
Selection of patients for data analysis. *N* = number of patients; *y* = years; 52 patients were excluded after analysing data because in most cases they were formally classified as “dying in ICU/IMC” but died either outside the ICU/IMC or, e.g., under CPR while admitted on ICU/IMC.

From January 2011 to December 2012, 1325 patients died in our hospital. Eight patients under 18 years old were excluded. From the remaining 1317 patients, 541 died either in an ICU or an IMC (Figure 1). We excluded 52 patients in most cases because they were formally classified as dying in an ICU/IMC but either died in the emergency room/operating room or reached the ICU under cardiopulmonary resuscitation (CPR). Finally, 489 data sheets of patients dying in an ICU/IMC in 2011 (267 patients) or in 2012 (222 patients) were analysed (Figure 1).

Patients' characteristics are shown in Table 1. About 70% (*n* = 352) of patients dying in an ICU/IMC in our hospital were older than 70 years. Most patients died in medical ICU/IMC, and most were treated because of cardiopulmonary problems. One patient labelled as pediatric was a 20-year-old disabled patient with a long medical history and treated for severe pneumonia in the anaesthetic/interdisciplinary ICU. Because declaration of religious affiliation in Germany is not mandatory, we could not extract this information from the data in many cases. Main diagnosis-related groups (DRG) of patients are shown in Table 1. Some patients are labelled as “intensive complex treatment” because the complexity of the intensive care treatment triggered a special reimbursement independent from the original medical problem. The cause of death as extracted from the official death certificate was assigned as cardial or pulmonary in almost half the patients followed by gastrointestinal causes or malignancies. Only few patients were classified as “died due to sepsis” because the cause of death was related to the main organ system; e.g., severe sepsis due to pneumonia is classified as pulmonary (Table 1). Time of death is distributed almost equally over time of the day and days of the week (Table 1). Only 57 patients (12%) had an advance directive. More than 80% of patients (*n* = 409) were less than 10 days in the ICU/IMC before they died, and almost 95% (*n* = 461) of patients died within less than 20 days in ICU/IMC. Most patients (75%, *n* = 372) were ventilated less than 100 hours before they died.

Table 2 measures at end-of-life versus sex, age and advance directive.

The different therapy measures at the end-of-life are shown in Table 2. About 60% of patients were ventilated, and about half of them had catecholamine therapy, received antibiotics, and were fed enterally or parenterally. Around 20% died under CPR or had CPR just before their death. Almost 80% received any kind of analgesia, and 37% got any kind of sedation drugs.

In about 60% of patients (*n* = 297), we found direct documentation or unambiguous hints for withholding of at least one life-sustaining measure (Table 3). In 45% existed a written “do not resuscitate” order. Invasive ventilation (24.7%), noninvasive ventilation (8.2%), renal replacement therapy (14.5%), catecholamines (13.5%) and other drugs, or feeding components were withheldless often.

In contrast to 297 patients with therapy withholding, we found withdrawal of any component of life-sustaining therapy in only 139 patients (=28.4%, Table 4). The fractions decreased from 22.9% for “other drugs” to 11.6% for catecholamine therapy, 10.4% for feeding, and in less than 5% of patients eitherrenal replacement therapy (4.5%), noninvasive (1.0%) or invasive ventilation (2.7%) was actively reduced or stopped.

**Figure 2:**
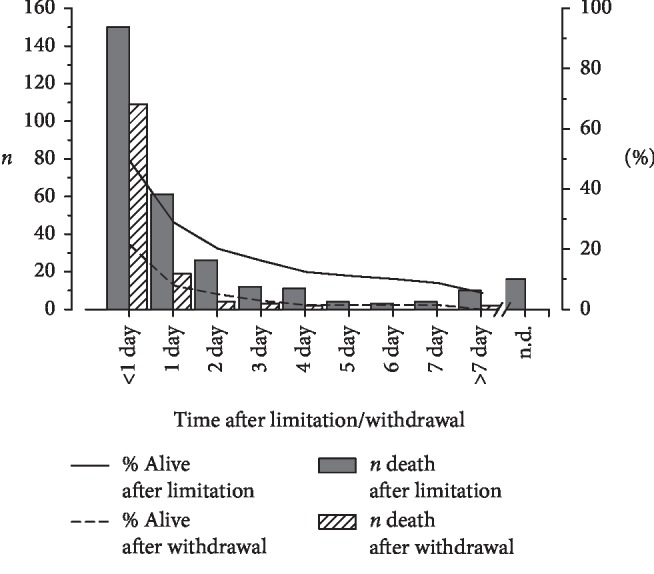
Time course after withholding or withdrawal of a life-sustaining therapy. The number of patients dying after withholding or withdrawal of a life-sustaining therapy is shown in bars. The percentage of patients who are still alive after withholding or withdrawal of therapy are plotted as lines.

After therapy withholding, half of the patients died within one day, and about 70% of percents died within two days (Figure 2). When life-sustaining therapy was withdrawn, about 80% of patients died within one day, and more than 90% of patients died within two days.

Table 4 withdrawal of therapy versus sex, age and advance directive.

There was no relevant difference in the frequency of the different measures at end-of-life as well as in the frequency of withholding or withdrawal between male and female patients (Tables 2–4). By trend, in younger patients, invasive measures are used more often and limited less often. As an example, patients invasively ventilated were younger than patients those not (70.5 ± 13.0 years vs. 77.6 ± 11.6 years, *p* < 0.05) as well as patients receiving catecholamine therapy (71.1 ± 12.8 years vs. 76.3 ± 12.5 years, *p* < 0.05) were younger than those who did not get catecholamines. In contrast, there was no difference in the age of patients receiving CPR or not before death (71.7 ± 13.0 years vs. 74.3 ± 12.8 years, *p*=0.95), and patients noninvasively ventilated were even older that patients who were not (79.0 ± 9.5 years vs. 73.5 ± 13.0 years, *p* < 0.05). There was no obvious relationship between withdrawal and age, but there was hardly any withdrawal at all in patients younger than 40 years (Table 4). Patients with a documented withholding of therapy were older (76.4 ± 11.4 years) than patients without (69.8 ± 13.7 years, *p* < 0.05, Table 3). In contrast, there was no difference in age between patients with (73.9 ± 12.4 years) or without (73.8 ± 13.1 years; *p*=0.97, Table 4) withdrawal of therapy.

We did not see any obvious difference in the frequency of use of the different therapy measures in therapy-withholding or withdrawal in patients of religious affiliation (data not shown). However, in most patients, the religious background is unknown, and absolute numbers of Jehovah's witnesses, Muslims, or “other” religions are very small.

Only 11.7% of patients dying in ICU/IMC had an advanced directive (Table 2). Those patients were older (80.1 ± 8.1 years, *n* = 57) than patients without one (73.0 ± 13.2 years, *p* < 0.05, *n* = 432). Patients with a written advance directive were less often ventilated invasively and had much less cardiopulmonary resuscitation (Table 2). By trend, therapy was more often limited and withdrawn when patients had an advance directive (Table 2).

## 4. Discussion

We investigated end-of-life decisions in adult ICU/IMC patients in an urban nonuniversity tertiary hospital. This is in contrast to multicenter evaluations like the ETHICUS study [19] or a large French study [26]. But in the ETHICUS study, only two German hospitals contributed data and both were large university hospitals [19]. Other studies investigating end-of-life decisions in German ICUs are from an University hospital, too [22, 23]. Because only 35 of 1942 hospitals in Germany are university hospitals [27], there is still too little known about end-of-life decisions in Germany.

In 2011/2012, we did not use a standardised documentation of therapy withholding/withdrawal. Only cases with clear documentation or unambiguous hints for withholding or withdrawal were classified as withheld or withdrawn. We might have missed cases of withholding/withdrawal which were done in agreement with the patient and/or the family but not explicitly mentioned in the chart. The “advanced directives law” (enacted in Germany in September 2009 [28]) states that physicians as well as relatives are obliged to respect patients' wishes/preferences referring to medical therapies. In our study, only about 12% of patients had a written advance directive which is comparable to low rates reported in other studies [22, 23]. We suppose that, in most cases, end-of-life decisions are medical decisions which have been discussed with patients or relatives to respect patient's wishes.

Time intervals as time from ICU arrival to death or duration of ventilation are probably reasonably exact because they are extracted from SAP®, checked by trained controllers and are the base for the refunding system in Germany. Unfortunately, due to paper-based documentation without exact time markers, an exact definition of withholding or withdrawal “time point” was not possible in many cases. But the relevant time intervals for analysis of time intervals after withholding or withdrawal are probably days and not hours (Figure 2).

About 70% of deceased patients in our study are older than 70 years, and almost 40% are even older than 80 years which is comparable to other investigations [19, 22, 23, 26]. It can be expected that the number of elderly patients in hospital and in ICUs will even further increase due to the demographic changes in the population [3]. Actually, there are more than 30 million people ≥65 years in Germany, and it is expected that this number even increases within the next decades [3] It is likely that the patient's age might influence end-of-life decisions because intensivists might be less reluctant to limit or even withdraw a therapy in older patients. Most patients died due to cardiopulmonary problems (Table 1) which again is similar to results in other investigations [22, 23, 26, 29].

End-of-life decisions differ in patients with different religious affiliation [19, 30] and in medical staff with different religious backgrounds [19, 30]. In most cases, the religious affiliation remained unknown because declaration of religious affiliation of hospital patients was voluntary. Surprisingly, the number of Muslims in our study is quite low because the Muslim population in Hanau is estimated to be about 20% of the overall population [31]. It cannot be excluded that many Muslim patients are “hidden” in the “unknown” group.

The even distribution of death might be due to the shift system which guarantees the presence of an ICU fellow in the ward and the presence of an intensive care consultant in the hospital 24 hours per day. Our colleagues from Berlin did not find a significant difference between weekdays and weekends, and they showed that most end-of-life decisions were done during normal working hours [22].

We could not analyse the causes of death in more detail. In Germany, there is no routine examination of patients dying in hospital by a coroner. Rarely, there is a postmortem section by a pathologist, and it cannot be excluded that the “cause of death” mentioned in the official death certificate is not always an exact diagnosis [32].

Therapy measures at end-of-life in our study are comparable but slightly different to that described in other studies [19, 22, 23, 26]. About 60% of patients were ventilated compared to 88.6% in the ETHICUS investigation [19] and around 80% in the study from Charité Hospital [22]. Similarly, we saw lower rates of patients receiving catecholamine therapy (47.9%) compared to around 60% in the other two studies [19, 22]. The lower rates of these two invasive measures might be due to the inclusion of IMC patients in our study, whereas other studies investigated ICU patients only [19, 22, 23, 26]. We included IMC patients because sometimes there is smooth transition between ICU and IMC therapy [33].

Around 17% of our patients died under CPR or had CPR just before their death (Table 2). This might be a hint that these patients died under continued maximal therapy. On the other hand, we categorized three patients as “dying under CPR,” although we found a “DNR” mark in their patients' charts. It is difficult to argue about the plausibility of CPR just by looking at the charts, but it is well known that many patients receive nonbeneficial treatment at the end-of-life [10]. Meanwhile, we introduced standard forms for end-of-life decisions in our hospital [34] to reduce imprudent transfers of patients to the ICU in emergency situations.

Almost 80% of deceased patients received any kind of analgesia, and 37% got any kind of sedation drugs. This is reassuring but one-fifth of dying patients did not receive analgesia, and almost 2/3 of dying patients did not get any sedation drug. We cannot say if patients without analgetics just did not have any pain or if it was forgotten to ask for pain and to subscribe an analgesic.

In about 60% of patients, we found withholding of at least one invasive measure (Table 3), and in about 30% of patients, at least one measure was withdrawn (Table 4) which was in the range reported [19, 22, 35]. The more invasive the measure, the more likely was a withholding of its use. It was more likely that the less-invasive measures such as drugs and catecholamines were withdrawn than renal replacement therapy or invasive ventilation. It can only be speculated that physicians are still more reluctant to withdraw a therapy if it is more invasive and they expect a prompt correlation between withdrawal and death. We observed a tendency that invasive measures are more often used and less often withheldin younger patients. However, for patients under 40 years old, the absolute number for “therapy measures”, and for patients under 60 years old, the absolute numbers for “therapy withholding” and “therapy withdrawal” are very low (Tables 3 and 4). It is likely that older patients might “get their chance” in the ICU, but doctors are more reluctant to initiate or to continue invasive measures.

The short time between withholding/withdrawal of therapy and death is expected because those therapy measures are–by definition–life sustaining. However, therapy is withheld/withdrawn just a few days before death, and we cannot exclude that invasive measures have already been applied to patients for too long [10].

Other investigators could show that early or regular involvement of a palliative care team member can reduce invasiveness of therapy in critically or terminally ill patients [16, 36–38]. We try to implement principles of good palliative care in our daily intensive care [39], but currently we do not check regularly for palliative care or end-of-life issues when admitting a patient to the ICU. We interact closely with the ambulant palliative care team in Hanau, and many physicians of our team have been working in the palliative care team for six to twelve months.

## 5. Conclusion

Withholding and/or withdrawal of therapy preceded most deaths in our ICU/IMC. By trend, more invasive measures were used less often but more often withheld in older patients. Most patients died within two days after withholding or withdrawal of a life-sustaining therapy.

## Figures and Tables

**Table 1:** Characteristics of patients dying in the ICU/IMC in 2011 and 2012. Numbers are shown as absolute numbers *n* and percentage (%) of all patients dying in ICU/IMC in 2011 and 2012.

Patients' characteristics		*n*	%
Sex	Male	266	54.4
	Female	233	47.6

Age (years)	18–29	4	0.8
	30–39	5	1.0
	40–49	19	3.9
	50–59	34	7.0
	60–69	75	15.3
	70–79	163	33.3
	80–89	161	32.9
	≥90	28	5.7

Speciality	Cardiology/pneumology	257	52.6
	Visceral/thoracic surgery	93	19.0
	Gastroenterology	39	8.0
	Vascular surgery	31	6.3
	Trauma/orthopaedic surgery	26	5.3
	Neurology	22	4.5
	Hemato oncology	19	3.9
	Gynaecology	1	0.2
	Paediatrics	1	0.2

Ward	Medical ICU	225	46.0
	Surgical ICU/IMC	179	36.6
	Medical IMC	85	17.4

Religion	Unknown	245	50.1
	Protestant	141	28.8
	Roman Catholic	87	17.8
	Without religious affiliation	5	1.0
	Jehovah's witnesses	4	0.8
	Muslim	4	0.8
	Others	3	0.6

Main diagnosis	Cardiovascular	135	27.6
	Intensive care complex treatment	97	19.8
	Respiratory system	61	12.4
	Gastrointestinal	45	9.2
	Urology	27	5.5
	Infectious, parasitic diseases	26	5.3
	Hepatobiliar, pancreatic	22	4.5
	Musculoskeletal	22	4.5
	CNS	21	4.3
	Hemato-oncology	12	2.4
	Endocrinology	8	1.6
	Dermatology	3	0.6
	Severely, multiple injured	3	0.6
	Others	2	0.4
	Hematology	2	0.4
	Eye	1	0.2
	Gynaecology	1	0.2
	Intoxication	1	0.2

Weekday of death	Monday	76	15.5
	Tuesday	71	14.5
	Wednesday	68	13.9
	Thursday	72	14.7
	Friday	69	14.1
	Saturday	71	14.5
	Sunday	62	12.7

Day-time/hour of death	0–4	77	15.7
	4–8	64	13.1
	8–12	81	16.6
	12–16	75	15.3
	16–20	92	18.8
	20–24	100	20.4

Days in the IMC/ICU	0–9	409	83.6
	10–19	52	10.6
	20–29	12	2.5
	30–39	7	1.4
	40–49	4	0.8
	50–59	2	0.4
	60–69	2	0.4
	70–79	1	0.2

Hours ventilated	0	169	34.6
	1–99	203	41.5
	100–199	49	10.0
	200–299	16	3.3
	300–399	16	3.3
	400–499	9	1.8
	500–599	6	1.2
	600–699	7	1.4
	700–799	3	0.6
	800–899	2	0.4
	900–999	4	0.8
	≥ 1000	5	1.0

**Table 2:** Therapy measures at end-of-life versus sex, age, and advance directive. Numbers are shown as absolute numbers *n* and percentage (%) of all patients dying in ICU/IMC or as percentage of patients in the subgroup, e.g., all female patients or all patients between 50 and 59 years old.

			Measures at end-of-life												
		*n*	IV	NIV	RRT	Catecholamines	Invasive BPM	CVL	CPR	Analgesia	Sedation	Antibiotic therapy	Diuretic therapy	Other drugs	Feeding nutrition
	Yes	489	260	26	48	234	315	318	85	387	179	263	199	361	268
			53.2%	5.3%	9.8%	47.9%	64.4%	65.0%	17.4%	79.1%	36.6%	53.8%	40.7%	73.8%	54.8%

Sex	Male	266	145	13	30	130	170	168	57	202	96	140	105	193	144
			54.5%	4.9%	11.3%	48.9%	63.9%	63.2%	21.4%	75.9%	36.1%	52.6%	39.5%	72.6%	54.1%
	Female	223	115	13	18	104	145	150	28	185	83	123	94	168	124
			51.6%	5.8%	8.1%	46.6%	65.0%	67.3%	12.6%	83.0%	37.2%	55.2%	42.2%	75.3%	55.6%

Age (years)	18–29	4	3	0	0	2	3	4	1	3	2	1	1	4	2
			75.0%	0.0%	0.0%	50.0%	75.0%	100.0%	25.0%	75.0%	50.0%	25.0%	25.0%	100.0%	50.0%
	30–39	5	4	0	0	4	4	4	1	4	3	4	1	5	2
			80.0%	0.0%	0.0%	80.0%	80.0%	80.0%	20.0%	80.0%	60.0%	80.0%	20.0%	100.0%	40.0%
	40–49	19	15	0	6	13	16	16	5	11	8	9	5	12	9
			78.9%	0.0%	31.6%	68.4%	84.2%	84.2%	26.3%	57.9%	42.1%	47.4%	26.3%	63.2%	47.4%
	50–59	34	24	1	3	19	25	25	7	27	15	22	12	26	18
			70.6%	2.9%	8.8%	55.9%	73.5%	73.5%	20.6%	79.4%	44.1%	64.7%	35.3%	76.5%	52.9%
	60–69	75	47	4	9	40	54	55	13	59	32	44	29	55	45
			62.7%	5.3%	12.0%	53.3%	72.0%	73.3%	17.3%	78.7%	42.7%	58.7%	38.7%	73.3%	60.0%
	70–79	163	102	5	20	95	119	122	30	135	74	92	71	128	83
			62.6%	3.1%	12.3%	58.3%	73.0%	74.8%	18.4%	82.8%	45.4%	56.4%	43.6%	78.5%	50.9%
	80–89	161	60	13	10	54	83	81	27	123	42	79	69	110	92
			37.3%	8.1%	6.2%	33.5%	51.6%	50.3%	16.8%	76.4%	26.1%	49.1%	42.9%	68.3%	57.1%
	≥90	28	5	3	0	7	11	11	1	25	3	12	11	21	17
			17.9%	10.7%	0.0%	25.0%	39.3%	39.3%	3.6%	89.3%	10.7%	42.9%	39.3%	75.0%	60.7%

Advance directive?	Yes	57	17	5	5	18	31	30	1	50	11	32	24	39	32
			29.8%	8.8%	8.8%	31.6%	54.4%	52.6%	1.8%	87.7%	19.3%	56.1%	42.1%	68.4%	56.1%
	No	432	243	21	43	216	284	288	84	337	168	231	175	322	236
			56.3%	4.9%	10.0%	50.0%	65.7%	66.7%	19.4%	78.0%	38.9%	53.5%	40.5%	74.5%	54.6%

Note: “n. expl. m.” refers to “not explicitly mentioned;” IV, invasive ventilation; NIV, noninvasive ventilation; RRT, renal replacement therapy; BPM, blood pressure measurement; CVL, central venous line; CPR, cardiopulmonary resuscitation; and DNR: do not resuscitate.

**Table 3:** Therapy withholding at end-of-life versus sex, age and advance directive. Numbers are shown as absolute numbers *n* and percentage (%) of all patients dying in ICU/IMC or as percentage of patients in the subgroup, e.g., all female patients or all patients between 50 and 59 years old.

			Therapy withholding?						
		*n*	Withholding?		IV	NIV	RRT	Catecholamines	DNR
	Yes	489	297	Yes	121	40	71	66	222
					24.7%	8.2%	14.5%	13.5%	45.4%
			60.7%	n. expl. m.	77	87	156	141	68
					15.7%	17.8%	31.9%	28.8%	13.9%
	No		192		291	362	262	282	199
			39.3%		59.5%	74.0%	53.6%	57.7%	40.7%

Sex	Male	266	147	Yes	58	19	36	32	113
					21.8%	7.1%	13.5%	12.0%	42.5%
			55.3%	n. expl. m.	40	44	75	68	30
					15.0%	16.5%	28.2%	25.6%	11.3%
	Female	223	150	Yes	63	21	35	34	109
					28.3%	9.4%	15.7%	15.2%	48.9%
			67.3%	n. expl. m.	37	43	81	73	38
					16.6%	19.3%	36.3%	32.7%	17.0%

Age (years)	18–29	4	1	Yes	1	1	0	0	1
					25.0%	25.0%	0.0%	0.0%	25.0%
			25.0%	n. expl. m.	0	0	1	1	0
					0.0%	0.0%	25.0%	25.0%	0.0%
	30–39	5	1	Yes	0	0	0	0	1
					0.0%	0.0%	0.0%	0.0%	20.0%
			20.0%	n. expl. m.	1	1	1	1	0
					20.0%	20.0%	20.0%	20.0%	0.0%
	40–49	19	6	Yes	2	0	2	2	6
					10.5%	0.0%	10.5%	10.5%	31.6%
			31.6%	n. expl. m.	0	0	2	3	0
					0.0%	0.0%	10.5%	15.8%	0.0%
	50–59	34	17	Yes	2	0	2	4	10
					5.9%	0.0%	5.9%	11.8%	29.4%
			50.0%	n. expl. m.	3	3	10	7	6
					8.8%	8.8%	29.4%	20.6%	17.6%
	60–69	75	40	Yes	18	6	14	11	32
					24.0%	8.0%	18.7%	14.7%	42.7%
			53.3%	n. expl. m.	5	5	15	16	4
					6.7%	6.7%	20.0%	21.3%	5.3%
	70–79	163	92	Yes	38	10	19	22	73
					23.3%	6.1%	11.7%	13.5%	44.8%
			56.4%	n. expl. m.	19	26	46	39	18
					11.7%	16.0%	28.2%	23.9%	11.0%
	80–89	161	113	Yes	49	16	26	20	81
					30.4%	9.9%	16.1%	12.4%	50.3%
			70.2%	n. expl. m.	36	38	64	58	31
					22.4%	23.6%	39.8%	36.0%	19.3%
	≥90	28	27	Yes	11	7	8	6	18
					39.3%	25.0%	28.6%	21.4%	64.3%
			96.4%	n. expl. m.	13	14	17	16	9
					46.4%	50.0%	60.7%	57.1%	32.1%

Advance directive?	Yes	57	52	Yes	20	8	14	9	38
					35.1%	14.0%	24.6%	15.8%	66.7%
			91.2%	n. expl. m.	18	21	31	27	13
					31.6%	36.8%	54.4%	47.4%	22.8%
	No	432	245	Yes	101	32	57	57	184
					23.4%	7.4%	13.2%	13.2%	42.6%
			56.7%	n. expl. m.	59	66	125	114	55
					13.7%	15.3%	28.9%	26.4%	12.7%

**Table 4:** Withdrawal of therapy versus sex, age and advance directive. Numbers are shown as absolute numbers *n* and percentage (%) of all patients dying in ICU/IMC or as percentage of patients in the subgroup, e.g. all female patients or all patients between 50 and 59 years old.

			Therapy withdrawal						
		n	Withdrawal?	IV	NIV	RRT	Catecholamines	Feeding nutrition	Other
Drugs	Yes	489	139	13	5	22	57	51	112
			28.4%	2.7%	1.0%	4.5%	11.7%	10.4%	22.9%
	No		350	476	484	467	432	377	438
			71.6%	97.3%	99.0%	95.5%	88.3%	77.1%	89.6%

Sex	Male	266	74	8	3	10	29	29	63
			27.8%	3.0%	1.1%	3.8%	10.9%	10.9%	23.7%
	Female	223	65	5	2	12	28	22	49
			29.1%	2.2%	0.9%	5.4%	12.6%	9.9%	22.0%

Age (years)	18–29	4	1	0	1	0	0	0	1
			25.0%	0.0%	25.0%	0.0%	0.0%	0.0%	25.0%
	30–39	5	0	0	0	0	0	0	0
			0.0%	0.0%	0.0%	0.0%	0.0%	0.0%	0.0%
	40–49	19	6	2	0	0	1	3	6
			31.6%	10.5%	0.0%	0.0%	5.3%	15.8%	31.6%
	50–59	34	10	3	0	2	4	6	7
			29.4%	8.8%	0.0%	5.9%	11.8%	17.6%	20.6%
	60–69	75	28	3	1	6	11	9	23
			37.3%	4.0%	1.3%	8.0%	14.7%	12.0%	30.7%
	70–79	163	36	1	1	9	16	15	28
			22.1%	0.6%	0.6%	5.5%	9.8%	9.2%	17.2%
	80–89	161	51	4	1	5	23	17	41
			31.7%	2.5%	0.6%	3.1%	14.3%	10.6%	25.5%
	≥90	28	7	0	1	0	2	1	6
			25.0%	0.0%	3.6%	0.0%	7.1%	3.6%	21.4%

Advance directive?	Yes	57	23	2	1	2	10		8
			40.4%	3.5%	1.8%	3.5%	17.5%		14.0%
	No	432	116	11	4	20	47		43
			26.9%	2.5%	0.9%	4.6%	10.9%		10.0%

Note: “n. expl. m.” refers to “not explicitly mentioned;” IV, invasive ventilation; NIV, noninvasive ventilation; RRT, renal replacement therapy; and DNR, do not resuscitate.

## Data Availability

The datasets used and/or analysed during the current study are available from the corresponding author upon reasonable request.

## Abbreviations

ICU: Intensive care unit
IMC: Intermediate care unit
DRG: German diagnosis-related group
CPR: Cardiopulmonary resuscitation
IV: Invasive ventilation
NIV: Noninvasive ventilation
RRT: Renal replacement therapy
BPM: Blood pressure measurement
CVL: Central venous line
CPR: Cardiopulmonary resuscitation
DNR: Do not resuscitate.
